# Seizure-induced hilar ectopic granule cells in the adult dentate gyrus

**DOI:** 10.3389/fnins.2023.1150283

**Published:** 2023-03-02

**Authors:** Yuka Kasahara, Hideyuki Nakashima, Kinichi Nakashima

**Affiliations:** Department of Stem Cell Biology and Medicine, Graduate School of Medical Sciences, Kyushu University, Fukuoka, Japan

**Keywords:** epilepsy, neuroinflammation, adult neurogenesis, ectopic neurogenesis, E/I balance

## Abstract

Epilepsy is a chronic neurological disorder characterized by hypersynchronous spontaneous recurrent seizures, and affects approximately 50 million people worldwide. Cumulative evidence has revealed that epileptogenic insult temporarily increases neurogenesis in the hippocampus; however, a fraction of the newly generated neurons are integrated abnormally into the existing neural circuits. The abnormal neurogenesis, including ectopic localization of newborn neurons in the hilus, formation of abnormal basal dendrites, and disorganization of the apical dendrites, rewires hippocampal neural networks and leads to the development of spontaneous seizures. The central roles of hilar ectopic granule cells in regulating hippocampal excitability have been suggested. In this review, we introduce recent findings about the migration of newborn granule cells to the dentate hilus after seizures and the roles of seizure-induced ectopic granule cells in the epileptic brain. In addition, we delineate possible intrinsic and extrinsic mechanisms underlying this abnormality. Finally, we suggest that the regulation of seizure-induced ectopic cells can be a promising target for epilepsy therapy and provide perspectives on future research directions.

## Introduction

**FIGURE 1:**
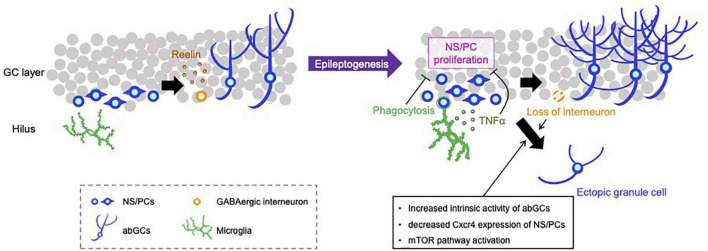
Representative negative and positive regulations for aberrant neurogenesis in the hippocampus. NS/PCs in the hippocampal subgranular zone proliferate and differentiate into GCs. Reelin released from GABAergic interneurons supports the integration processes of abGCs in the healthy brain **(left)**. SE induces an increase in the number of proliferating NS/PCs and subsequent depletion of NS/PCs, appearance of hilar ectopic GCs, mossy fiber sprouting, increased dendritic sprouting and loss of inhibitory interneurons **(right)**. Activated microglia phagocytose live newborn neurons and suppress the emergence of ectopic GCs. Microglia also attenuate the proliferation of NS/PCs by TLR9-mediated secretion of TNF-α, resulting in the reduction of aberrant neurogenesis. Loss of GABAergic neurons and intracellular alteration of abGCs properties, e.g., increased intrinsic neuronal activity, downregulation of Cxcr4 expression and activation of the mTOR pathway, can promote the mis-migration of abGCs to the hilus.

Epilepsy is a diverse group of neurological disorders characterized by excessive hypersynchronous discharge-induced seizures. The hippocampal dentate gyrus (DG) has been implicated in the development of epilepsy due to its unique circuitry (Jessberger and Parent, 2015; Danzer, 2019). The DG is the primary gating structure of the hippocampus, and its circuitry establishes an inhibitory feedback circuit comprised of interneuron microcircuits and regulates the flow of excitatory input from the cortex essential for spatial learning and memory (Sahay et al., 2011). Accumulated evidence has indicated that neural stem/progenitor cells (NS/PCs) are retained even in the adult subgranular zone of the DG (Eriksson et al., 1998; Boldrini et al., 2018; Terreros-Roncal et al., 2021; Zhou et al., 2022) and they proliferate and give rise to new neurons throughout life in a process referred to as adult neurogenesis (Gross, 2000; Gage, 2019). Adult-born granule cells (abGCs) are integrated into the existing mature brain networks and exhibit age-dependent effects on hippocampal network activity (Danielson et al., 2016; McHugh et al., 2022), contributing to brain plasticity and maintenance of proper brain functions. However, in the DG of temporal lobe epilepsy (TLE) animal models and patients, accelerated aberrant proliferation of NS/PCs, abnormal abGC migration, formation of hilar basal dendrites and mossy fiber sprouting are observed, resulting in the aberrant integration of abGCs into the preexisting neural circuits (Jessberger and Parent, 2015; Danzer, 2019). This abnormal integration is thought to disrupt the dentate gate and increase the excitability of the hippocampus circuitry, leading to perpetuation of recurrent seizures (Parent et al., 1997; Overstreet Wadiche et al., 2005; Zhang et al., 2012; Zhou et al., 2019). Mapping analysis of large neuronal population dynamics has revealed that seizures are not simply recurrent bursts of hypersynchrony. Instead, it is becoming clear that seizures involve a complex interplay of different neural cell populations and circuits (Bui et al., 2015; Sparks et al., 2020). Based on computational modeling studies it is suggested that a network in which a small number of dentate GCs are hyperconnected to each other (hub network) is more effective at promoting seizure-like activity than a network wherein all dentate GCs are more interconnected than the control state (Morgan and Soltesz, 2008). Recent studies revealed that abGCs localized in the dentate hilus, hilar ectopic GCs, can play a role in the epileptic hub network (Scharfman and Pierce, 2012; Althaus et al., 2019; Lybrand et al., 2021). In this review, we discuss recent evidence demonstrating the characteristics and roles of hilar ectopic GCs in the epileptic brain and describe cell-intrinsic and extrinsic mechanisms underlying their mis-migration (Figure 1). Finally, we also offer some perspectives on future research directions.

## Hilar ectopic granule cells

Prolonged seizure activity promotes NS/PC proliferation for several weeks after epileptogenic insult (Parent et al., 1997; Scott et al., 2000) and newborn GCs generated from these NS/PCs are integrated into neural networks heterogeneously (Murphy et al., 2011). Under healthy conditions, GCs exhibit a typical morphology: a round cell body located in the GC layer, fanlike dendritic trees extending to the molecular layer, and an absence of basal dendrites (Claiborne et al., 1990). By contrast, under epileptic conditions, a substantial fraction of newborn GCs migrate ectopically into the dentate hilus and the molecular layer and develop aberrant properties such as acquisition of hilar basal dendrites, mossy fiber sprouting, and increased dendritic arborization (Parent et al., 1997; Jessberger et al., 2007; Figure 1). A subpopulation of newborn GCs forms abnormal connections with neighboring GCs *via* sprouted mossy fibers or aberrant basal dendrites (Murphy et al., 2011; Du et al., 2017). Because abGCs exert a hippocampal network-level modulatory role throughout their maturation (McHugh et al., 2022), alterations in the network structure of abGCs may explain the effects on hippocampal excitability in the epileptic brain.

Hilar ectopic GCs are a pathological hallmark commonly observed in animal models and patients with TLE (Parent et al., 2006; Pierce et al., 2011). The abGCs are particularly vulnerable to epileptogenic insults. To determine the precise developmental stages at which GCs migrate ectopically, Kron et al. (2010) conducted cell birthdate studies in an experimental epilepsy model. They injected retroviral reporters to label dividing progenitor cells and suppressed neurogenesis by x-irradiation at specific times 2–4 weeks before or 4 days after pilocarpine-induced status epilepticus (SE) leading to the development of epilepsy. Only cells born post-SE were significantly more likely to migrate into the hilus (around 20% of labeled cells) and a fraction of the newborn GCs displayed hilar basal dendrites and mossy fiber sprouting (around 30% of labeled cells). Moreover, the study of Singh et al. (2015) clarified whether ectopic GCs arose ubiquitously throughout the NS/PC pool or were derived from a more restricted NS/PC subpopulation. They performed a clonal analysis study in mice expressing Brainbow fluorochromes in individual glioma-associated oncogene homolog1-expressing type 1 progenitor cells and identified a specific fraction of progenitors that produce the majority of ectopic GCs in response to an epileptogenic insult (Singh et al., 2015). Progenitor cells producing hilar ectopic GCs appear to generate only ectopic cells, whereas those producing the cells in the appropriate position in the dentate continued to do so. In epileptic conditions, the affected progenitors or their local microenvironments may become pathological, driving hilar ectopic cell migration. Alternatively, the affected progenitor cells may be functionally normal but misled to areas where migratory cues have been distorted, driving the generation of ectopic cells.

Open questions in the field include whether and how hilar ectopic cells contribute to network hyperexcitability leading to the development of epilepsy. To address this question, several groups have scrutinized features of the ectopic cells including the potential for burst firing and altered excitation/inhibition (E/I) ratios (Scharfman et al., 2000; Zhan and Nadler, 2009; Zhan et al., 2010; Althaus et al., 2015). Recently, Althaus et al. (2019) investigated the relationship between GCs’ birthdate, morphology, and network integration in a pilocarpine TLE model. By recording spontaneous excitatory and inhibitory currents, they found that both early-born (P7) and adult-born (P60) populations of GCs received increased-excitatory input after the seizure, compared with age-matched sham controls. When abGCs were divided into normally integrated (normotopic) and aberrant (ectopic or hilar basal dendrites-containing) subpopulations, only the aberrant populations showed a relative increase in excitatory input. The ratio of excitatory-to-inhibitory input was most dramatically upregulated in hilar ectopic GCs, which implicates the ectopic GCs as drivers for network hyperexcitability in epileptic conditions. The authors hypothesized that these aberrantly integrated cells act as “hub cells” for initiating or propagating seizure activity. The diversity among the abGCs population highlights a key problem of experiments aiming to manipulate specific newborn GC subtypes in epilepsy. A study using an inducible transgenic alteration of mammalian target of rapamycin (mTOR) signaling to disrupt the normal development of a fraction of early-born GCs revealed that the generation of abnormal GCs including ectopic GCs is enough to induce spontaneous seizures (Pun et al., 2012). In support of this, pharmacogenetic suppression of the post-seizure aberrant neurogenesis including the generation of the ectopic GCs reduced the later development of spontaneous seizures (Cho et al., 2015; Hosford et al., 2016). Furthermore, chemogenetic silencing of abGCs reduced the number of ectopic GCs and seizure occurrence (Lybrand et al., 2021). However, it should be noted that reducing neurogenesis has not always been found to mitigate epilepsy development. Although Zhu et al. (2017) decreased abnormal integrations of abGCs into the neural circuit *via* the ablation of cells with methylazoxymethanol acetate, they found no effect on the development of spontaneous seizures in a pilocarpine model. Brulet et al. (2017) used a genetic approach to reduce neurogenesis by deleting the transcription factor *NeuroD1* gene in NS/PCs prior to SE. This conditional *NeuroD1* deletion indeed reduced abnormal integrations; however, seizure frequency did not change after pilocarpine administration (Brulet et al., 2017). These inconsistent results could be attributed to side effects of the drug, potential toxic effects of systemic antimitotic drugs, and/or insufficient reductions of ectopic GCs. The efficacy of manipulation of neurogenesis on seizure development may also depend on the time-point and targeted cell populations. It is conceivable that approaches targeting only abnormal cells would be more effective and broadly applicable, and would enable us to elucidate the role of hilar ectopic cells in the development of epilepsy. In the next section, we describe intrinsic and extrinsic mechanisms that help us to develop strategies to specifically manipulate hilar ectopic cells.

## Cell-intrinsic mechanisms regulating the emergence of hilar ectopic granule cells

Accumulating evidence suggests that neuronal activity regulates adult neurogenesis (Kempermann et al., 2015; Denoth-Lippuner and Jessberger, 2021) and improper migration of abGCs has been linked to increased intrinsic neuronal activity (Sim et al., 2013). Recently, Lybrand et al. (2021) investigated whether activity during the maturation of immature abGCs plays a critical role to drive aberrant alterations of cellular behavior including ectopic migration. To manipulate the intrinsic activity of abGCs at different stages of maturation, proliferating NS/PCs were first infected with an excitatory designer receptor (hM3Dq)-expressing retrovirus and the synthetic ligand clozapine-N-oxide (CNO) was injected once daily to activate abGCs for the first (0–1 w) or second (1–2 w) week after retroviral infection. Activation during both the first and second periods of maturation promoted abnormal migration of abGCs into the hilus without dendrite developmental change. When the labeled 8 w-old mature GCs were activated with CNO, no effects on migration or cell morphology were observed. These results suggest that a critical time window during which neuronal activity is associated with aberrant maturation exists and activity during this period seems sufficient to promote the migration of abGCs to the hilus. Zero–1w and 1–2w activation provoked spontaneous recurrent seizures at 8 weeks post-infection in 60 and 80% of hM3Dq-virus injected mice, respectively. In the pilocarpine model, chemogenetically silencing immature aberrant abGCs through an inhibitory designer receptor (hM4Di) during this critical period reduced ectopic cells, abnormal dendritic morphology, and the occurrence of spontaneous seizures. They also found that 2w-old abGCs exhibited dynamic calcium fluctuations, and stimulation of both designer receptors (hM3Dq and hM4Di) could modulate the intracellular calcium of immature abGCs. These observations imply that cellular intrinsic activity *via* calcium response regulates the migration of newborn cells appropriately, but it is unclear how sustained elevation of intrinsic calcium levels affects abGCs’ development and maturation. In mouse cerebellar granule cells, the amplitude and frequency of calcium transients, *via* voltage-gated calcium channels (VGCCs), are correlated positively with the rate of neuronal migration, suggesting that calcium acts as a speedometer to integrate various intrinsic/extrinsic cues that drive neuronal migration (Komuro and Rakic, 1996). Although the role of VGCCs in immature abGCs is poorly defined, it has been reported that VGCCs generate low-threshold somatic calcium spikes in immature abGCs before 3 weeks of age, which are trophic cues that promote neuronal development (Konur and Ghosh, 2005). In addition, enhanced VGCCs currents with altered properties occur in the dentate GCs of epileptic patients (Jeub et al., 1999; Djamshidian et al., 2002). These results suggest that increased VGCCs currents in immature abGCs promote improper cellular integration into existing dentate GCs circuits, causing the development of epilepsy (Figure 1). Downstream regulators of calcium signaling in aberrant neurogenesis could also be potential targets for anti-epileptic drugs.

We have previously shown that prenatal exposure to valproic acid (VPA), which is known to function as an antiepileptic drug by inhibiting, e.g., gamma-aminobutyric acid (GABA) transaminase, leads to the reduction of adult neurogenesis and increase of the malpositioned GCs in the dentate hilus (Sakai et al., 2018). These effects were paired with an increase in the susceptibility to kainic acid (KA) -induced seizures in adulthood. To identify the mechanism underlying the accumulation of ectopic GCs, we performed RNA sequencing analysis and found that CXC motif chemokine receptor 4 (Cxcr4) expressed in the prenatally VPA-exposed NS/PCs was downregulated in the adult. Overexpression of *Cxcr4* selectively in NS/PCs using a retroviral strategy attenuated the ectopic migration of abGCs and seizure susceptibility. Although it has been reported that *Cxcr4* deletion consistently reduces adult neurogenesis and leads to the appearance of ectopic GCs (Schultheiß et al., 2013), how and why Cxcr4 expression has such an effect is not fully understood. One possible explanation is that *Cxcr4* overexpression restores the migratory cues needed for immature GCs to correctly integrate into the GC layer, reducing ectopic migration. VPA increases GABA levels in the brain and GABA can bind to and activate Cxcr4 (Guyon et al., 2013). Indeed, it was suggested that Cxcr4 ligand Cxcl12 is probably co-secreted with GABA from hippocampal interneurons, and thus they both may possibly affect NS/PC behavior (Bhattacharyya et al., 2008). By disrupting GABA and/or Cxcl12 signaling in the neurogenic niche, VPA may interfere with the Cxcr4-regulated migration of immature GCs. Another possibility is that VPA induces changes in the expression of many genes as an epigenetic drug because it has histone deacetylase inhibitor activity (Hsieh et al., 2004; Xu et al., 2007) that positively regulates gene expression by promoting histone acetylation. Understanding the contribution of epigenetic-mediated gene regulation may reveal new regulatory mechanisms of cell migration.

The mTOR pathway, which regulates neuronal migration, growth, and plasticity, is activated in several models of epilepsy (Zeng et al., 2009; Huang et al., 2010). Phosphatase and tensin homolog (PTEN) is a lipid phosphatase that targets the 3′ phosphate of phosphatidylinositol 3,4,5 triphosphate, acting in opposition to phosphatidylinositol 3-kinase (PI3K). mTOR is a major target in the PI3K pathway and deletion of PTEN leads to excess activation of mTOR (Kwon et al., 2003). Deletion of PTEN in postnatally produced GCs led to an increase in ectopically located cells, causing spontaneous seizures (Pun et al., 2012). PTEN-deficient GCs also recapitulate numerous other morphological pathologies associated with TLE, including mossy fiber sprouting, hypertrophy, the appearance of basal dendrites, and increased dendritic spine density (Kwon et al., 2001; Pun et al., 2012). After KA administration, treating animals with the mTOR antagonist rapamycin mitigates GC aberrant migration and mossy fiber sprouting, which directly links the mTOR pathway to these phenomena (Shima et al., 2015). mTOR signaling is involved in the regulation of GC dispersion and migration deeper into the GC layer closer to the molecular layer boundary (Getz et al., 2016). It is worthwhile noting that the mechanisms regulating the appearance of abnormal cells in two discrete locations, the hilus and the molecular layer, can be distinct.

## Cell-extrinsic mechanisms regulating the emergence of hilar ectopic granule cells

Reelin, an extracellular matrix glycoprotein, plays an essential role in neuronal migration and the formation of laminated brain structures, such as the neocortex, hippocampus, and cerebellum (Chai and Frotscher, 2016). After cortical development and the demise of most Cajal-Retzius cells, Reelin is expressed primarily by interneurons in the adult brain (Lee and D’Arcangelo, 2016). Immunohistochemical studies revealed that the cell bodies of Reelin-expressing interneurons were located within the subgranular adult stem cell niche. These Reelin-expressing cells were identified as cholecystokinin-positive but not parvalbumin-positive cells (Pahle et al., 2020). Disorganized GC layer formation has been shown to be accompanied by a loss of Reelin-producing neurons in the epileptic hippocampus (Haas et al., 2002; Heinrich et al., 2006; Orcinha et al., 2016). In the DG of Reelin-deficient *reeler* mice, the GCs are scattered, suggesting a GC migration defect (Katsuyama and Terashima, 2009). Antibody-blockade of Reelin function in naive mice causes dispersion of GCs, and exogenous administration of Reelin after epileptogenic injury prevents the dispersion (Haas and Frotscher, 2010). Consistently, conditional knockout of the disabled-1 gene (*Dab1*), encoding an adaptor protein that is essential for Reelin signaling, in adult or postnatal NS/PCs results in abnormal migration, such that new granule neurons are scattered throughout the DG area (Teixeira et al., 2012; Korn et al., 2016). Both *reeler*- and *Dab1*-deficient mice lack spontaneous seizures but exhibit enhanced seizure susceptibility (Patrylo et al., 2006; Korn et al., 2016). In contrast, interneuron-specific Reelin knockout did not cause structural changes in the DG (Pahle et al., 2020). In this mouse, the number of Reelin-expressing Cajal-Retzius cells, which can function compensatorily for the loss of Reelin-expressing interneurons, increased, eventually resulting in the formation of an organized GC layer. However, it has become apparent that Reelin’s functions are not simple. Using hippocampal slice culture and live imaging, Wang et al. (2018) revealed that Reelin acts as an attractant dictating the migration of GCs toward the molecular layer. In addition, Reelin has also been suggested to function as a local repulsing cue to ensconce mature GCs in the normotopic position, suppressing GC’s aberrant migration under epileptic conditions (Orcinha et al., 2021).

Gamma-aminobutyric acid can directly control neurogenesis, and disruption of GABA homeostasis triggers abnormal neurogenesis (Tozuka et al., 2005; Nakamichi et al., 2009). In the DG, the primary sources of GABA are inhibitory interneurons, i.e., parvalbumin- and somatostatin-neurons. Immature dentate GCs express high levels of sodium, potassium, chloride cotransporter 1 (NKCC1), a cation-Cl^–^ importer, which changes the reversal potential for Cl^–^, causing GABA_A_ receptor activation to depolarize the cell (Ge et al., 2006). Immature abGCs initially receive the depolarizing GABA signal, which is necessary for their proper development (Overstreet Wadiche et al., 2005). A recent study showed that GABA-mediated amplification of intracellular calcium regulates the early critical period of activity associated with the aberrant maturation of abGCs (Lybrand et al., 2021). Treatment with the GABA_A_ receptor agonist phenobarbital increases hilar ectopic GCs in normal rat pups and conversely, treatment with the GABA_A_ receptor antagonist picrotoxin decreased it in a postnatal febrile seizure (which is thought to be a triggering insult for TLE) model (Koyama et al., 2012; Kasahara et al., 2019). Following epileptogenic insults, GABAergic interneurons are overstimulated and presumably die *via* excitotoxicity, and surviving interneurons may compensate for this loss and become hyperexcitable abnormally (Kobayashi and Buckmaster, 2003; Wang et al., 2016). In hilar ectopic GCs, GABA_A_ receptor signaling mediates tonic GABA currents that occur after SE (Zhan and Nadler, 2009). Increased depolarizing effect of GABA on immature abGCs would impose a hyperexcitable signal on mature dentate GCs and enhance hippocampal excitability. To enhance GABA’s hyperpolarizing effect on mature neurons and attenuate hippocampal hyperexcitability, medial ganglionic eminence-derived GABAergic neuronal progenitor cells were grafted into the hippocampus and its antiepileptic effect was demonstrated (Hunt et al., 2013; Cunningham et al., 2014; Upadhya et al., 2019). Recently, Arshad et al. (2022) reported that following transplantation of GABAergic neuronal progenitors, ectopic immature abGCs decreased in epileptic model mice, while normotopic immature abGCs increased in not only the epileptic model but also in intact mice. These effects on migration/localization of abGCs could be due to Reelin released from the transplanted interneurons. Alternatively, modifying the E/I balance in the hippocampus may affect abGCs integration.

It has become clear from considerable evidence that glial cells modulate adult neurogenesis in the hippocampus (Cope and Gould, 2019). Microglia have been shown to regulate each step of adult neurogenesis, such as proliferation, survival, and maturation of newly generated cells both in the non-epileptic and epileptic brains (Luo et al., 2016a). We have previously shown that microglia suppress aberrant neurogenesis by inhibiting the hyper-proliferation of NS/PCs after KA-induced seizures through the activation of Toll-like receptor 9 (TLR9) in microglia (Matsuda et al., 2015). In the epileptic condition, microglia sense self-DNA, which is presumably released from degenerating neurons, *via* TLR9 and then secrete TNF-α, and TNF-α in turn attenuates hyper-proliferation of NS/PCs. After KA injection, GCs localized in the hilus increased in TLR9-deficient mice, and infusion of recombinant TNF-α into the ventricle reduced the hilar ectopic GCs. Furthermore, TLR9 deficiency exacerbated seizure-induced cognitive decline and recurrent seizure severity. These findings indicate that activated microglia reduce abnormally located newborn neurons through the secretion of TNF-α, resulting in antiepileptic effects. Microglia can also directly regulate the emergence of ectopic GCs by phagocytosis. In the healthy brain, microglia mainly engulf apoptotic abGCs, but after SE, activated microglia switch their target and prefer to engulf caspase-negative live abGCs (Luo et al., 2016b). Luo et al. (2016b) reported that the administration of minocycline, an inhibitor of microglial activation, reduced the number of engulfed newborn GCs, increasing the number of ectopic GCs. These results suggest that microglia suppress the appearance of excess newborn cells and eliminate abGCs after SE to inhibit the formation of abnormal neural circuits leading to the development of epilepsy. In contrast to these results, some studies reported that genetic ablation of microglia or suppression of microglial activation with minocycline reduced immature neurons, suggesting that microglia accelerate seizure-induced neurogenesis (Ali et al., 2015; Mo et al., 2019). Yang et al. (2010) demonstrated that after pilocarpine-induced SE, suppression of microglial activation with minocycline reduced the number of ectopic GCs. These discrepancies regarding the role of microglia in neurogenesis after SE may be attributable to differences in experimental settings to mimic epilepsy-like phenotypes. Indeed, microglia show different gene expressions depending on the types of chemoconvulsants, i.e., KA, and pilocarpine (Benson et al., 2015). In addition, “activated” microglia exhibit heterogeneity in their properties, which makes it difficult to corroborate the precise roles of microglia in adult neurogenesis.

Astrocytes also play roles in the proliferation and neuronal fate commitment of NS/PCs in the adult hippocampus (Song et al., 2002), and regulation of synapse integration into existing neural circuits through astrocytic vesicular release (Sultan et al., 2015). These findings suggest that astrocytes are important regulators of adult neurogenesis at all stages of the process. In the DG of the epileptic patient, astrocytes are activated and the length of astrocytic fibers around the GC layer significantly increases (Fahrner et al., 2007; Thom, 2014). However, whether and how astrocytes regulate aberrant adult neurogenesis in the epileptic brain remains largely unknown. Further studies are necessary to clarify the contribution of astrocytes to the aberrant neurogenesis characteristic of the epileptic brain.

## Conclusion

Here, we review findings about seizure-induced ectopic GCs in animal models and humans with epilepsy. It is becoming clear that ectopic localization of abGCs in the dentate hilus radically alters the types of afferent inputs and efferent outputs, and that these ectopic cells exhibit hyperexcitable features. A key question that remained to be answered is whether ectopic GCs are the major player which rewires neural networks, accounting for hippocampal hyperexcitability, the characteristic of the epileptic brain. Several studies using distinct techniques have commonly demonstrated that targeted ablation of abGCs including hilar ectopic GCs can significantly reduce seizures, suggesting that abGC-specific manipulations could be beneficial for epilepsy treatment. However, there exist reports suggesting that abGCs ablation exacerbates epilepsy development, warranting further investigation to clearly elucidate the functional implications of hilar ectopic GCs acting as hub-like cells in the epileptic DG networks. Given the heterogeneous properties of abGCs after SE, populations of normotopically and ectopically integrated abGCs are likely to have opposing effects on neuronal excitability. Although current techniques for ablating or silencing abGCs affect all abGCs regardless of their subtypes, characterization and identification of the subtypes will enable us to target the specific hilar GC subtype for the therapeutic treatment of epilepsy. Elucidating the cellular and molecular mechanisms underlying seizure-induced ectopic abGCs would shed light on our understanding of brain plasticity attributable to adult neurogenesis. Taking all of these considerations together, we believe that seizure-induced hilar ectopic GCs represent a promising target for intervention in the treatment of human epilepsy and its comorbidities.

## Author contributions

YK, HN, and KN: conceptualization and writing—review and editing. YK and HN: writing—original draft. All authors contributed to the article and approved the submitted version.
